# Increased 1,25(OH)_2_-Vitamin D Concentrations after Energy Restriction Are Associated with Changes in Skeletal Muscle Phenotype

**DOI:** 10.3390/nu13020607

**Published:** 2021-02-12

**Authors:** Angela Vidal, Rafael Rios, Carmen Pineda, Ignacio Lopez, Ana I. Raya, Escolastico Aguilera-Tejero, Jose-Luis L. Rivero

**Affiliations:** 1 Department of Animal Medicine and Surgery, University of Cordoba, 14071 Cordoba, Spain; v92vicaa@uco.es (A.V.); rafariosvaro@me.com (R.R.); v32pimac@uco.es (C.P.); l02lovii@uco.es (I.L.); v82rabea@uco.es (A.I.R.); 2 Maimonides Biomedical Research Institute of Cordoba (IMIBIC), Reina Sofia University Hospital, University of Cordoba, 14004 Cordoba, Spain; 3 Department of Comparative Anatomy, Pathological Anatomy, and Toxicology, University of Cordoba, 14071 Cordoba, Spain; an1lorij@uco.es

**Keywords:** energy restriction, muscle, vitamin D, rat

## Abstract

The influence of energy restriction (ER) on muscle is controversial, and the mechanisms are not well understood. To study the effect of ER on skeletal muscle phenotype and the influence of vitamin D, rats (*n* = 34) were fed a control diet or an ER diet. Muscle mass, muscle somatic index (MSI), fiber-type composition, fiber size, and metabolic activity were studied in tibialis cranialis (TC) and soleus (SOL) muscles. Plasma vitamin D metabolites and renal expression of enzymes involved in vitamin D metabolism were measured. In the ER group, muscle weight was unchanged in TC and decreased by 12% in SOL, but MSI increased in both muscles (*p* < 0.0001) by 55% and 36%, respectively. Histomorphometric studies showed 14% increase in the percentage of type IIA fibers and 13% reduction in type IIX fibers in TC of ER rats. Decreased size of type I fibers and reduced oxidative activity was identified in SOL of ER rats. An increase in plasma 1,25(OH)_2_-vitamin D (169.7 ± 6.8 vs. 85.4 ± 11.5 pg/mL, *p* < 0.0001) with kidney up-regulation of CYP27b1 and down-regulation of CYP24a1 was observed in ER rats. Plasma vitamin D correlated with MSI in both muscles (*p* < 0.001), with the percentages of type IIA and type IIX fibers in TC and with the oxidative profile in SOL. In conclusion, ER preserves skeletal muscle mass, improves contractile phenotype in phasic muscles (TC), and reduces energy expenditure in antigravity muscles (SOL). These beneficial effects are closely related to the increases in vitamin D secondary to ER.

## 1. Introduction

Energy restriction (ER) has been shown to retard aging and to have multiple beneficial effects on health by modulating metabolism and preventing organ deterioration [1]. The effect of ER on skeletal muscle is controversial. Many studies have demonstrated a decrease in skeletal muscle mass after ER, which is explained by activation of metabolic pathways that shift metabolism from anabolism to catabolism and by the use of muscle proteins as a source of energy [2,3]. However, other reports have shown that ER may have benefits on skeletal muscle, particularly in preventing age-related loss of muscle mass [4,5,6,7]. A variety of mechanisms have been proposed to explain the beneficial effects of ER on muscle, including: retarding deterioration of neuromuscular function [8], diminishing the dysfunction of slow muscles [9], modifying mitochondrial ultrastructure [10], decreasing mitochondrial oxidative damage [11], and increasing skeletal muscle insulin sensitivity [12].

Vitamin D is an essential micronutrient involved in bone and mineral metabolism. Vitamin D is synthesized in the skin or ingested with the diet as cholecalciferol, which subsequently is metabolized in the liver to 25(OH)-cholecalciferol (25(OH)-vitamin D). The 25(OH)-vitamin D is further hydroxylated in the kidney to produce 1,25(OH)_2_-cholecalciferol (1,25(OH)_2_-vitamin D), which is the major active metabolite of vitamin D [13].

In addition to its bone actions, vitamin D plays an important role in other body systems, including the skeletal muscle [14]. Several studies have found an association between vitamin D deficiency and myopathy or muscle weakness [15,16,17]. Moreover, many disorders that cause sarcopenia (e.g., aging, chronic kidney disease, obesity/metabolic syndrome) are also associated with vitamin D deficiency [18,19]. Furthermore, vitamin D supplementation has benefits in the treatment of sarcopenia, and adequate vitamin D status helps to prevent loss of muscle mass [20,21].

Vitamin D metabolism is regulated by fibroblast growth factor 23 (FGF23), a hormone produced by bone cells. FGF23 down-regulates 1,25(OH)_2_-vitamin D production by the kidney through the inhibition of the enzyme 1-α-hydroxylase (CYP27b1) that metabolizes 25(OH)-vitamin D to 1,25(OH)_2_-vitamin D and by stimulation of the catabolic enzyme 24-hydroxylase (CYP24a1) [22]. Recent reports demonstrate that low-calorie diets decrease FGF23 production, which in turn may result in increased 1,25(OH)_2_-vitamin D levels [23].

We hypothesized that an increase in 1,25(OH)_2_-vitamin D secondary to ER may play a role in the effects of ER on skeletal muscle. Thus, the objectives of this study were (1) to investigate the effect of ER on skeletal muscle histomorphometry and (2) to study the influence of ER on vitamin D metabolism and the relationship between vitamin D metabolites and changes in skeletal muscle mass and phenotype.

## 2. Materials and Methods

### 2.1. Animals and Diets

Animals were provided by the Animal Housing Facilities of the University of Cordoba (Cordoba, Spain). Thirty-four female Wistar rats aged two months at the beginning of the study were housed in individual cages with a 12:12 h light/dark cycle. Two diets with identical vitamin D concentration (500 IU/kg) were used in the experiments: control diet containing 3518 Kcal/Kg (Altromin C 1090-10, Altromin Spezialfutter GmbH, Lage, Germany) and a diet with a low caloric content, 1314 Kcal/kg (Altromin C 1012, Altromin Spezialfutter GmbH, Germany). Nutrient composition of both diets is shown in Table 1.

### 2.2. Experimental Design

Animals were randomly allocated to either a control group or an ER group (*n* = 17 each). Rats allotted to the control group were fed ad libitum the control diet, and rats in the ER group were fed the hypocaloric diet. Food was presented to the rats at 08:00. Since rats fed hypocaloric diet had increased appetite, in this group, food intake was adjusted in order to achieve 40% ER when compared with the control group. The experiments lasted 200 days. At the end of the study, rats were fasted for 12 h (food was withdrawn at 20:00) and sacrificed by exsanguination under general anesthesia (inhaled isoflurane, IsoVet, Braun, Spain) to obtain blood samples from the abdominal aorta and tissue samples from skeletal muscle, abdominal fat, and kidney. Abdominal fat was collected by removing the visceral fat surrounding the intestine and by scraping the inner surface of the abdominal wall.

### 2.3. Muscle Sampling

Muscles were obtained at the time of sacrifice. Tibialis cranialis (TC) and soleus (SOL) were chosen as representative muscles of fast-twitch and slow-twitch phenotypes, respectively. Both muscles were dissected and wet weighted. Muscle somatic index (MSI), used as a predictor of sarcopenia, was obtained by calculating the muscle weight (mg) to body weight at the time of sacrifice (g) ratio.

After collection, muscles were processed for histomorphometric studies as previously described [24]. Muscle samples were mounted on a cork block by using the Optimal Cutting Temperature (OCT) medium (Tissue-Tek II; Miles Laboratories, Naperville, IL, USA) so that the myofibers were oriented transversely. Thereafter, the samples were frozen by immersion in isopentane for 30 s and immediately transferred to liquid nitrogen to maintain the optimum freezing point. The processed muscles were stored at −80 °C until analyzed.

### 2.4. Histological Staining and Image Analysis

For histomorphometric studies, muscle samples (SOL and white region of TC) were serially cut in 10 µm sections with a cryostat (Frigocut; Reitchert Jung, Nubloch, Germany) at a working temperature of −20 °C. Two histochemical methods previously validated for rat skeletal muscle [25] were used for fiber-type identification: myofibrillar acid-ATPase (mATPase) and succinate dehydrogenase (SDH). These two stains quantify contractile (mATPase) and oxidative (SDH) enzyme activities. Stained sections were digitalized with Pinnacle Studio Software (Pinnacle Systems, version 24.0). A region containing approximately 150 fibers was chosen for morphological analysis using Scion Image Software version 4.0. All images were processed in a greyscale (1 to 255). To assess the oxidative profile, SDH activity was analyzed in optical density (OD) units in a 0 to 0.8 scale. The average fiber OD for the SDH histochemical reaction was determined as the average OD for all pixels within the traced fiber from three sections incubated with substrate (succinic acid) minus the average OD for all pixels of the same fiber from other two sections incubated without substrate. Because a number of factors can influence the reliability of histochemical enzyme activity determinations, the variability on three consecutive sections for the SDH histochemical reaction was checked by repeated measurements of the same individual fibers. Only coefficients of variation for triplicate measurements of ODs below 5% were accepted in the present study; this demonstrated the high analytical precision that can be achieved for the measurement of fiber OD on enzyme histochemical sections.

Since oblique orientation of fibers can occur when cutting muscle samples, both cross sectional area (CSA) and lesser fiber diameter were used to measure fiber size. Lesser fiber diameter is defined as the maximum diameter registered in the region where fiber shows the minor visual size and is designed to avoid the distortion that happens when fibers are not in transversal section. Each measurement was performed manually using the images from the SDH stain. Pixels were adjusted to micrometers with an appropriate calibrator.

Fiber phenotype was classified in one of the following types: type I, type IIA, type IIX, and type IIB. Hybrid phenotypes were distributed equally to one of its closer types. The percentage of each fiber-type was calculated for both muscles to analyze the fiber-type composition.

### 2.5. Blood Biochemistry

After blood collection, plasma was separated by centrifugation (3500 rpm for 10 min at 4 °C) and stored at −20 °C until assayed. ELISA test was used to quantify plasma intact FGF23 (Kainos Laboratories, Tokyo, Japan). Plasma levels of 25(OH)-vitamin D and 1,25(OH)_2_-vitamin D were determined by radioimmunoassay (Immunodiagnostic Systems Ltd., Boldon, UK). Assay performance was as follows: 25(OH)-vitamin D: specificity = 100% for 25(OH)-vitamin D_3_ and 75% for 25(OH)-vitamin D_2_; sensitivity = 1.2 ng/mL. 1,25(OH)_2_-vitamin D: specificity = 100% for 1,25(OH)-vitamin D_3_ and 91% for 1,25(OH)-vitamin D_2_; sensitivity = 2.1 pg/mL.

### 2.6. RNA Extraction and Real-Time RT-PCR

Kidney tissue was disrupted using liquid nitrogen and grinded thoroughly with a mortar. Total RNA was isolated using TRIzol reagent protocol (Invitrogen, Carlsbad, CA, USA), and a treatment with DNAse I amplification Grade (Sigma-Aldrich, St. Louis, MO, USA) was done according to the manufacturer’s instruction. Quantification was performed by spectrophotometry (ND1000, Nanodrop Technologies, Wilmington, DE, USA). Fifty nanograms of total RNA were used to analyze mRNA expression in the Light Cycler thermal cycler system (Roche Diagnostics, Indianapolis, IN, USA). QuantiTect SYBR Green RT-PCR kit (Qiagen GmbH, Hilden, Germany) was used for quantification following the manufacturer’s protocol. Results were normalized to GAPDH by using the 2^−ΔΔCt^ method. Primers for CYP27b1 and CYP24a1 quantification were purchased from Sigma Aldrich (Sigma-Aldrich St. Louis, MO, USA). Sequences for GAPDH were purchased from Eurofins (Eurofins Genomics, Germany GmbH, Ebersberg, Germany) (Table 2).

### 2.7. Statistics

Statistical analysis was performed using GraphPad Prism version 6.01 software (GraphPad Software, La Jolla, CA, USA). Values are expressed as mean ± standard error (SE). The difference between means was determined by Student’s t-test. Pearson’s test was used for correlation analysis. Differences between means were considered significant when *p* < 0.05.

## 3. Results

### 3.1. Energy Intake, Body Weight, Fat Mass, and Muscle Mass

**Figure 1:**
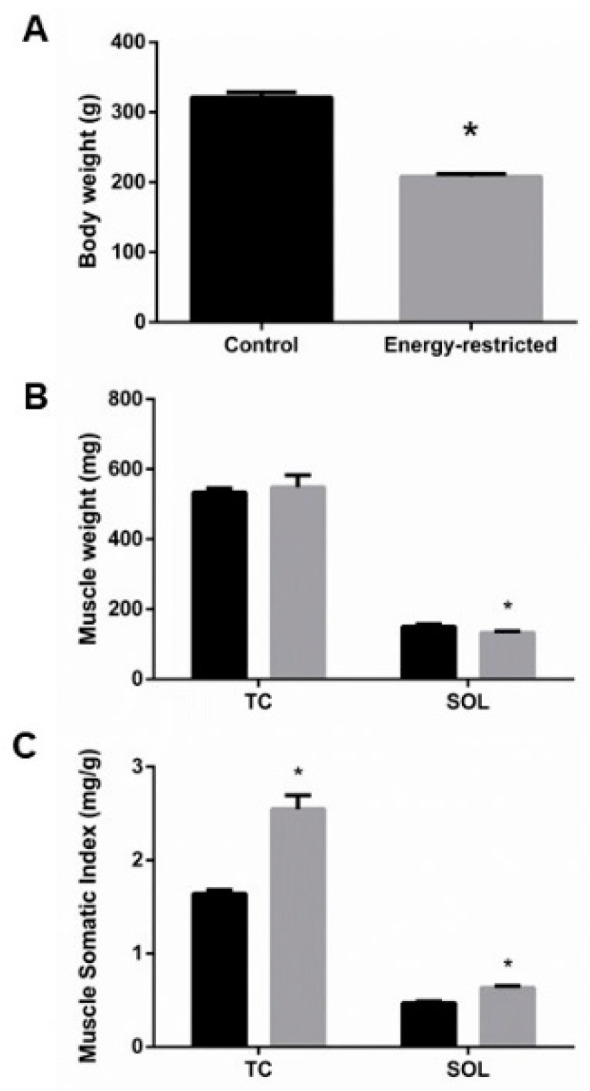
(**A**) Body weight at the time of sacrifice. (**B**) Tibialis cranialis (TC) and soleus (SOL) muscle weight, and (**C**) muscle somatic index. Student’s t-test between control (black bars) and energy-restricted (grey bars) groups. * *p* < 0.05 vs. control.

During the experiments, rats that ate hypocaloric diet lost 12.9% (from 239.0 ± 2.7 to 208.0 ± 3.7 g) of their weight, while control rats gained 31.3% (from 244.6 ± 1.9 to 321.3 ± 7.2 g) of their initial weight, *p* < 0.0001 (Figure 1A). Abdominal fat, weighted at the time of sacrifice, was much lower in ER rats, 1.7 ± 0.2 g, than in controls, 20.4 ± 2.4 g, *p* < 0.0001. Even though rats from the ER group were 35% lighter and had ~90% less abdominal fat than rats from the control group, only minor changes in muscle mass were found. ER did not decrease the weight of TC; however, SOL weighted 12% less in the ER group when compared to the control group, *p* = 0.03 (Figure 1B). MSI, which quantifies the muscle mass related to body weight, was significantly increased in both TC and SOL of rats fed the hypocaloric diet, 55% and 35.5%, respectively, *p* < 0.0001 (Figure 1C).

### 3.2. Muscle Histomorphometry

#### 3.2.1. Fiber-Type Composition

**Figure 2:**
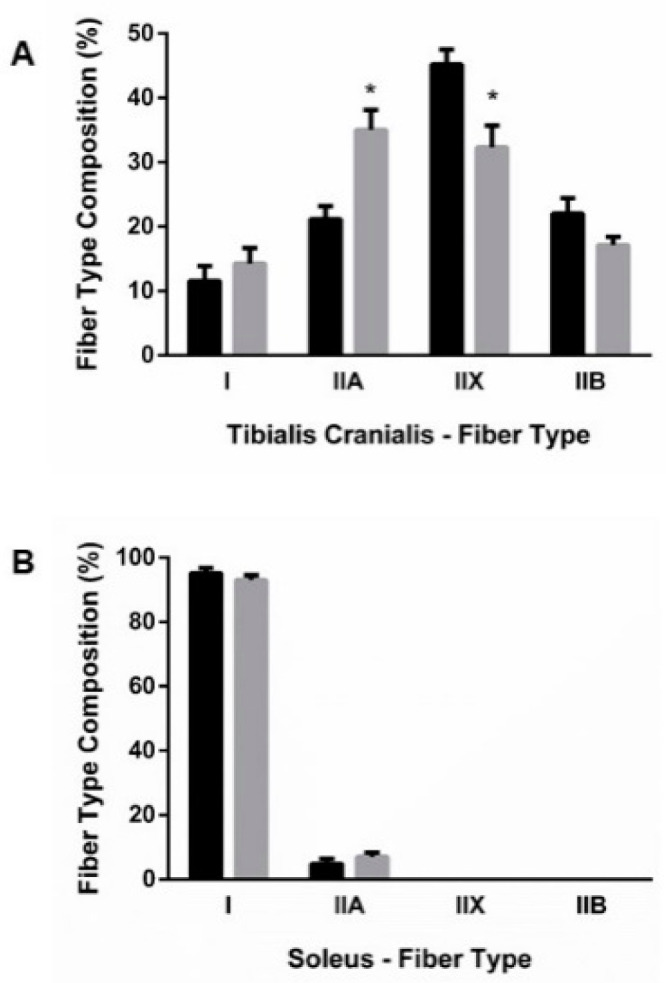
Fiber-type composition of (**A**) tibialis cranialis (TC) and (**B**) soleus (SOL) muscles. Student’s t-test between control (black bars) and energy-restricted (grey bars) groups. * *p* < 0.05 vs. control.

Four muscle fiber-types were identified in TC: types I, IIA, IIX, and IIB. As seen in Figure 2A, when compared with the control group, the TC of rats subjected to ER had a higher proportion of type IIA fibers that were increased by 13.8%, *p* = 0.001, while the percentage of type IIX fibers decreased by 12.8%, *p* = 0.006. ER did not modify the proportion of type I and type IIB fibers in TC. SOL was composed only of type I and type IIA fibers, with a clear predominance of type I fibers. A small reduction in the percentage of type I (2.2%) with a corresponding increase in the percentage of type IIA (2.2%) fibers was observed in SOL of rats from the ER group when compared to control rats (Figure 2B).

#### 3.2.2. Muscle Fiber Size

**Figure 3:**
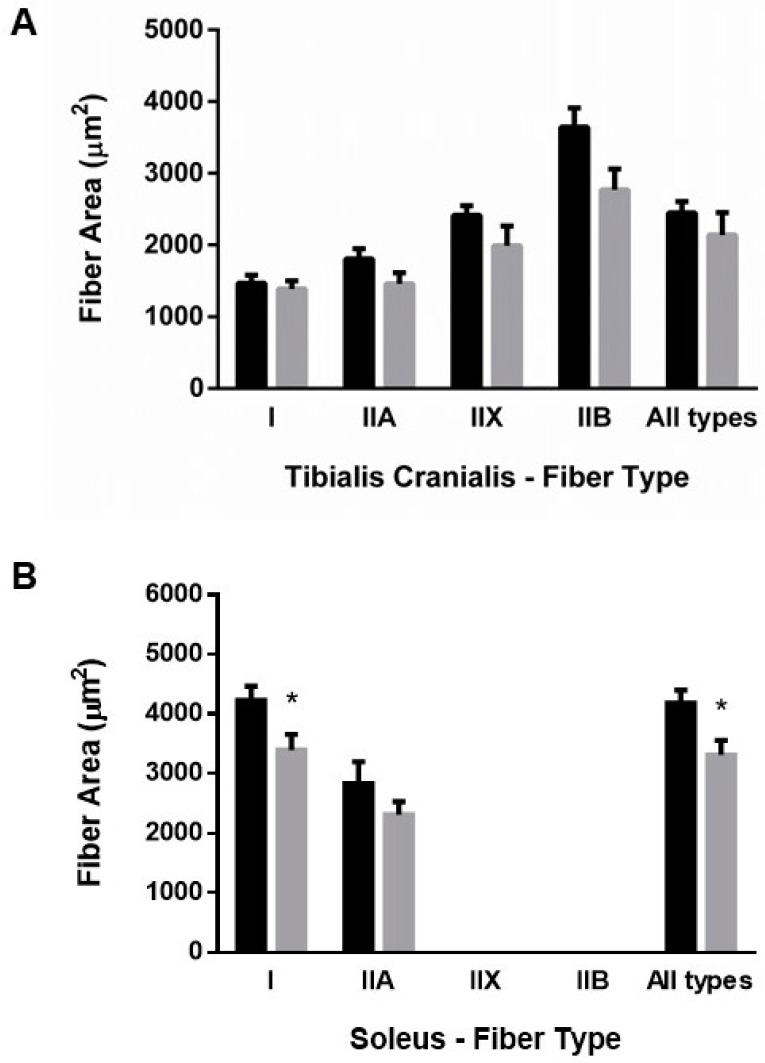
Mean fiber cross-sectional area of (**A**) tibialis cranialis and (**B**) soleus muscles. Student’s t-test between control (black bars) and energy-restricted (grey bars) groups. * *p* < 0.05 vs. control.

Excellent agreement was found between CSA and lesser fiber diameter, thus all results are reported as CSA. ER resulted in mild generalized atrophy of muscle fibers. In TC, atrophy was more evident in type II fibers than in type I, but the differences did not reach statistical significance in any type of fiber (Figure 3A). By contrast, muscle fiber CSA was significantly decreased in SOL of ER rats, 3313 ± 245 µm^2^, when compared with controls, 4181 ± 222 µm^2^, *p* = 0.015. This decrease in fiber size affected mainly type I fibers 3395 ± 260 vs. 4234 ± 234 µm^2^ in controls, *p* = 0.025 (Figure 3B).

#### 3.2.3. Muscle Oxidative Profile

**Figure 4:**
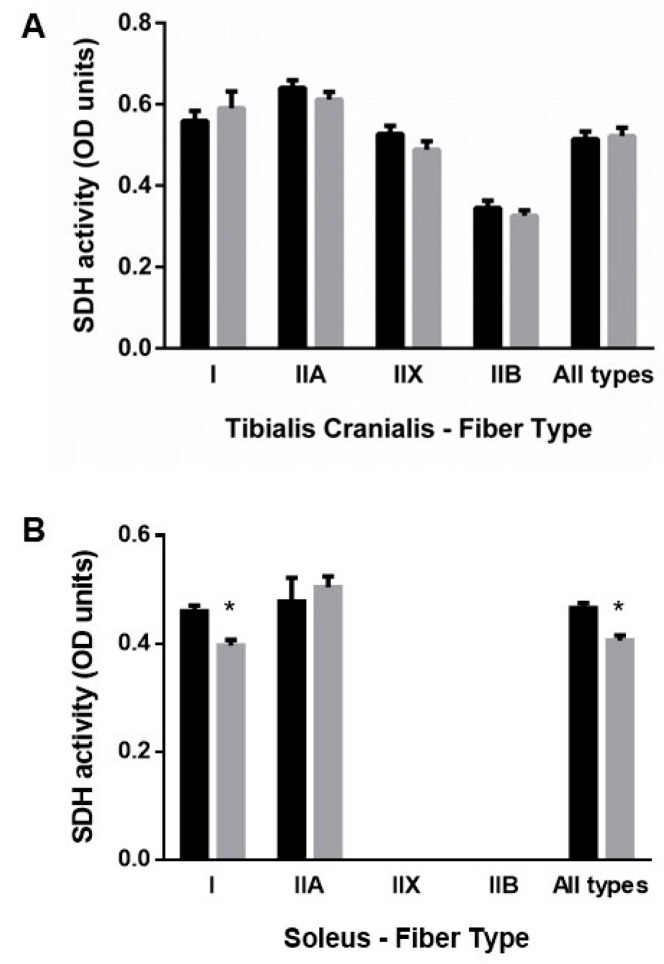
Mean fiber succinate dehydrogenase (SDH) activity in (**A**) tibialis cranialis and (**B**) soleus muscles. Student’s t-test between control (black bars) and energy-restricted (grey bars) groups. * *p* < 0.05 vs. control.

As shown in Figure 4A, the oxidative profile of muscle fibers, measured by SDH activity, did not change in TC. In contrast, in SOL, SDH activity was reduced in type I fibers of rats from the ER group, 0.41 ± 0.01 vs. 0.47 ± 0.01 OD units in the control group, *p* < 0.001 (Figure 4B).

### 3.3. Studies on Vitamin D and Related Metabolites

**Figure 5:**
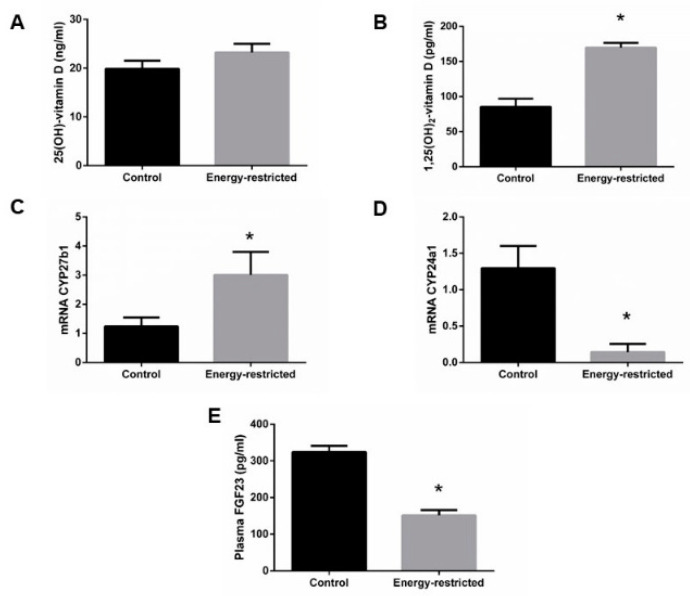
(**A**) Plasma concentrations of 25(OH)-vitamin D and (**B**) 1,25(OH)_2_-vitamin D, (**C**) kidney mRNA expression of the enzymes CYP27b1 and (**D**) CYP24a1 relative to GAPDH, and (**E**) plasma concentrations of fibroblast growth factor 23 (FGF23). Student’s t-test between control (black bars) and energy-restricted (grey bars) groups. * *p* < 0.05 vs. control.

Plasma 25(OH)-vitamin D concentration was not different between groups, 19.9 ± 1.7 vs. 23.2 ± 1.8 ng/mL, *p* = 0.181 (Figure 5A), but plasma 1,25(OH)_2_-vitamin D concentration was higher in energy-restricted rats, 169.7 ± 6.8 pg/mL, than in controls, 85.4 ± 11.5 pg/mL, *p* < 0.0001 (Figure 5B). The increase in 1,25(OH)_2_-vitamin D levels was consistent with the results of mRNA assays, which showed an increase in the renal expression (mRNA/GAPDH) of CYP27b1, 3.01 ± 0.78 vs. 1.24 ± 0.31, *p* = 0.027 (Figure 5C) and a decrease in the renal expression of CYP24a1, 0.14 ± 0.11 vs. 1.30 ± 0.31, *p* = 0.011 (Figure 5D) in ER rats. ER rats also had lower plasma concentrations of FGF23, 151.2 ± 14.1 pg/mL, than control rats, 324.5 ± 16.8 pg/mL, *p* < 0.0001 (Figure 5E).

**Figure 6:**
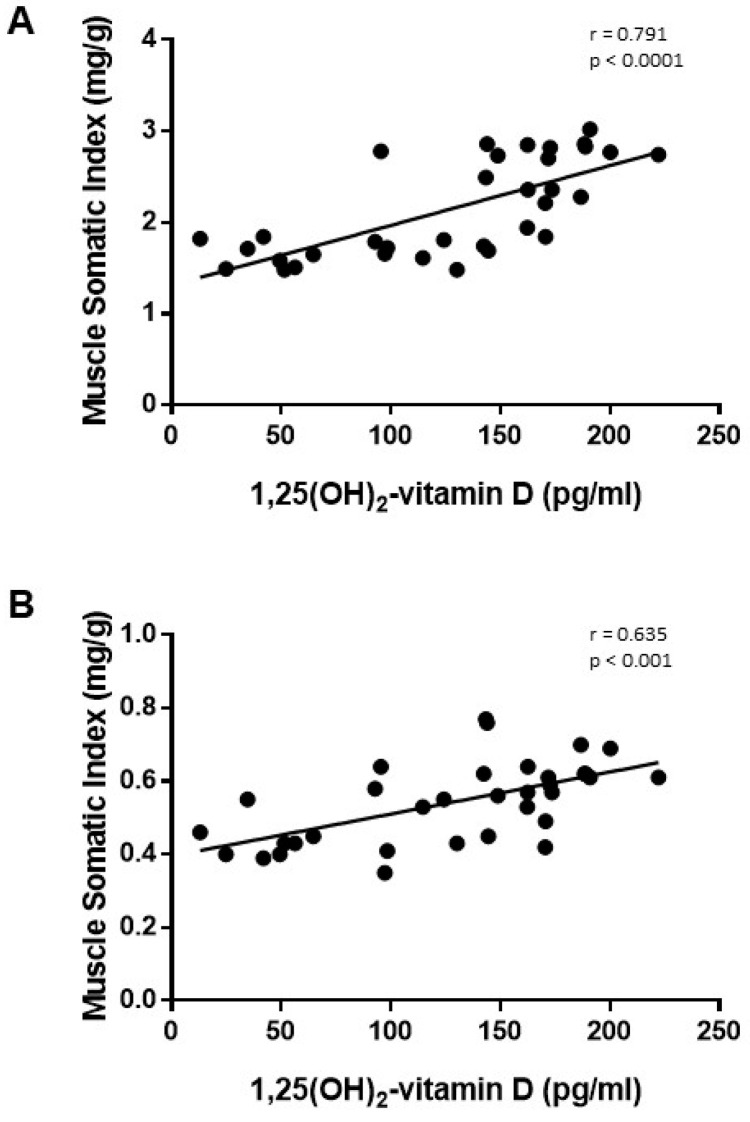
Correlation study (Pearson test) between plasma concentrations of 1,25(OH)_2_-vitamin D vs. muscle somatic index in (**A**) tibialis cranialis and (**B**) soleus muscles of control and energy-restricted rats.

The increase in plasma 1,25(OH)_2_-vitamin D was positively correlated with the increase in MSI observed in rats from the ER group in both muscles, TC and SOL (Figure 6). Changes of fiber-type composition in TC were also well correlated to plasma 1,25(OH)_2_-vitamin D concentration; the increase in 1,25(OH)_2_-vitamin D after ER was positively correlated with the percentage of type IIA fibers (r = 0.772, *p* < 0.001) and inversely correlated with the percentage of type IIX fibers (r = −0.638, *p* = 0.004). In addition, a significant inverse correlation between plasma 1,25(OH)_2_-vitamin D and SDH activity was observed in SOL (r = −0.491, *p* = 0.009).

## 4. Discussion

This study was designed to investigate the effect of ER on skeletal muscle phenotype and the influence of vitamin D on these changes. Our results demonstrate that, although ER promoted lack of continuous growth of postural muscles, the MSI was, in fact, increased. Moreover, after ER, fast-twitch muscles tended to evolve to a slower phenotype. These skeletal muscle changes were strongly correlated with the increase in plasma concentrations of 1,25(OH)_2_-vitamin D found in ER rats.

The protocols that are more widely used for ER in rodents involve a reduction of the total amount of food provided to the animals, either by decreasing daily food intake or, less frequently, by alternate day feeding [26,27]. These protocols not only reduce energy intake but also protein intake, which is essential for maintenance of muscle growth and function [28,29]. To avoid this limitation and to isolate the effect of energy intake on muscle, in the present study, rats were fed a hypocaloric diet that provided normal amount of proteins but reduced calories. In fact, in our effort to prevent protein restriction, ER rats ended up ingesting slightly more proteins than controls throughout the experiments (798 vs. 670 g, *p* < 0.05), and this may have had some partly anti-catabolic effect on muscle. Previous studies have shown that adequate intake of high-quality proteins is a factor in promoting muscle protein synthesis and preventing loss of muscle strength associated with aging [30].

The effect of ER on skeletal muscle mass is controversial. A number of studies have reported that ER reduces muscle weight and size [31,32,33,34], but this outcome has been shown to change over time and is not consistent in all muscles [33,35]. Our results agree with these reports since we observed a decrease in muscle mass in SOL but not in TC. The disparity may be explained by the function of each muscle. SOL is a postural muscle whose main function is to support body weight; therefore, the reduction of body weight after ER would decrease the stimulus for SOL growth [36], which was reflected in decreased muscle mass and decreased fiber size. These results are consistent with human studies that have shown decreased fiber size in women with low body weight [37]. This effect was not seen in TC, which is mainly used for locomotion and would be affected preferentially by physical activity [38]. All these data seem to indicate that the muscle atrophy observed after ER is more likely related to decreased body weight than to diminishing energy availability.

In agreement with other studies [33,39], we found an increase in the MSI of both muscles in ER rats. The increase in MSI was influenced by the decrease in body weight and more specifically by the decrease in body fat. However, these results cannot be considered a mathematical artifact because they reflect preservation of muscle mass in the face of severe ER. Moreover, the higher MSI should provide functional advantages [40,41].

For this study, we selected two muscles with different contractile and metabolic phenotypes, SOL and TC. SOL is a slow-twitch muscle mostly composed by type I fibers while TC is a fast-twitch muscle with a predominance of type II fibers. Phenotype of skeletal muscle is not static and can be modulated by different factors such as nutrition, exercise, or disease [42,43,44]. Phenotypic changes are more common in phasic muscles which participate in propulsion during the movement. After exercise and training, phasic muscles adapt to a slower contractile profile, which improves resistance and reduces the effects of muscle fatigue [45,46]. Likewise, the metabolic profile of the fibers also changes to preserve oxidative activity [47]. In this study, the rats on ER experienced a fast-to-slow switch in the fiber composition of TC without changes in the oxidative profile. The contractile phenotype of TC after ER was characterized by an increase in type IIA and a decrease in type IIX fibers as well as a tendency to increase type I fibers. Our results agree with previous reports demonstrating that ER ameliorates the effect of aging on muscle by reducing the percentage of type IIX and increasing type IIA fibers [39]. These changes are similar to the remodeling observed during training and are likely to improve muscle function [47,48]. In SOL, we did not find any significant changes in fiber type distribution, but oxidative profile was reduced in rats subjected to ER, and the decrease in SDH activity reached statistical significance in type I fibers. The decrease of this mitochondrial enzyme in SOL would be consistent with the need to support less body weight.

Rats subjected to ER had increased levels of 1,25(OH)_2_-vitamin D, and plasma concentrations of 1,25(OH)_2_-vitamin D showed a strong positive correlation with MSI both in TC and SOL. Although correlation does not always mean causation, vitamin D is known to be involved in skeletal muscle function and growth [49,50] and has been shown to induce hypertrophy of muscle fibers [51]. Thus, the increase in 1,25(OH)_2_-vitamin D levels found after ER is likely to produce anabolic effects on muscle. Vitamin D has been reported to influence muscle phenotype by modulating the number of type IIA fibers [50,52]. Interestingly, we found that the increase in the percentage of type II fibers after ER was strongly correlated to plasma 1,25(OH)_2_-vitamin D levels. These data are also in agreement with a recent study showing that 1,25(OH)_2_-vitamin D administration corrected the changes induced by uremia in the skeletal muscle of rats and increased the percentage of type IIA fibers [24].

The mechanisms by which 1,25(OH)_2_-vitamin D influences muscle phenotype have not been fully elucidated. The action of 1,25(OH)_2_-vitamin D on muscle is likely to be mediated by interaction with the vitamin D receptor (VDR). VDR over-expression has been reported to promote muscle anabolism [53], while VDR knockouts show muscle atrophy [54]. The 1,25(OH)_2_-vitamin D stimulates myogenesis through both genomic and non-genomic VDR-mediated mechanisms. Genomic actions involve interactions with vitamin D response element (VDRE), which has been found in many myogenic-related genes. Non-genomic mechanisms seem to affect mainly signal transduction in the ubiquitin–proteasomal pathway [55].

Previous work has demonstrated that energy intake directly regulates plasma FGF23 synthesis and secretion by bone cells. High caloric intake results in an increase in plasma FGF23, while reduced caloric intake decreases plasma FGF23 [23,56]. Our results showing lower FGF23 concentrations in the plasma of rats subjected to ER than in controls are consistent with these findings. FGF23 is a major regulator of vitamin D metabolism that inhibits the production of active vitamin D by modulating renal enzymes involved in 1,25(OH)_2_-vitamin D synthesis and catabolism. The data collected in the present study demonstrate that ER rats had significantly higher plasma concentrations of 1,25(OH)_2_-vitamin D than controls. No significant differences between controls and ER rats were found in the plasma concentrations of 25(OH)-vitamin D. Given that 25(OH)-vitamin D is the most reliable marker of dietary vitamin D status, any effect due to a difference in vitamin D intake between groups can be ruled out. However, ER resulted in a significant up-regulation of the main enzyme involved in 1,25(OH)_2_-vitamin D synthesis, CYP27b1, and a significant down-regulation of the main enzyme involved in 1,25(OH)_2_-vitamin D catabolism, CYP24a1. It is interesting to note that CYP24a1 was down-regulated even in the presence of elevated 1,25(OH)_2_-vitamin D, which, through interaction with kidney VDR, is known to up-regulate CYP24a1 [57]. Since these enzymes, CYP27b1 and CYP24a1, are directly regulated by FGF23, the increase in plasma 1,25(OH)_2_-vitamin D concentration seems to be secondary to the decrease in FGF23 production after ER.

Sequestration of vitamin D in adipose tissue [58] and volumetric dilution [59] have been proposed as mechanisms that may reduce vitamin D concentrations in obese people. Theoretically, the opposite, i.e., release from fat and volumetric concentration, could occur after ER. Although these mechanisms apply mostly to vitamin D and 25(OH)-vitamin D, they might also have some influence on circulating levels of 1,25(OH)_2_-vitamin D and could have contributed to the increased concentrations observed in ER rats.

**Figure 7:**
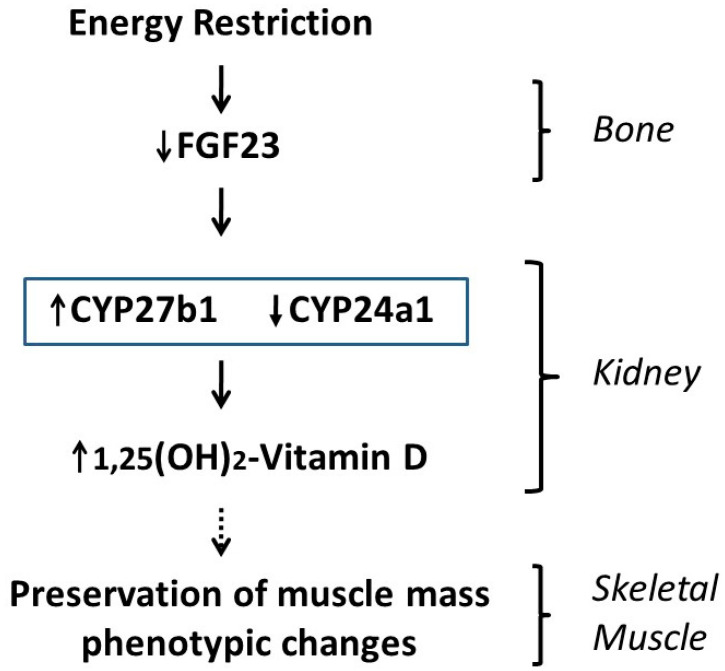
Proposed mechanism by which energy restriction modulates skeletal muscle mass and phenotype through regulation of vitamin D metabolism.

The proposed mechanisms, involving a bone–kidney–muscle axis by which ER may influence muscle mass and phenotype through vitamin D up-regulation, are summarized in Figure 7.

This study has some limitations. Since ER acts at many different levels, it is difficult to dissociate the effects of increased 1,25(OH)_2_-vitamin D from other influences of ER on muscle, such as modifications in mechanical load or changes in glucose metabolism/insulin sensitivity.

## 5. Conclusions

In conclusion, this study demonstrates that ER preserves skeletal muscle mass and modifies skeletal muscle fiber to a slower phenotype in phasic muscles (TC). These beneficial effects are closely related to the increases in 1,25(OH)_2_-vitamin D levels secondary to activation of the bone–renal axis after ER that subsequently may impact muscle.

## Figures and Tables

**Table 1:** Analytical composition of the two diets used in the study.

	Control	Energy Restriction
**Crude Nutrients (%)**		
Nitrogen free extractives	60	24.3
Crude protein	20.7	17.1
Crude fat	4	2
Crude fiber	3.1	44
Crude ash	4.3	5.5
Moisture	7.9	7.1
**Metabolized Energy (%)**		
Carbohydrates	66	34
Protein	24	52
Fat	10	14

**Table 2:** Sequences of primers used for RT-PCR in renal tissue.

Gene	Forward Primer (5′-3′)	Reverse Primer (5′-3′)
GAPDH	AGGGCTGCCTTCTCTTGTGAC	TGGGTAGAATCATACTGGAACATGTAG
CYP27b1	AAGAGTGATGACTACTGGG	ATAGTATCAAATAGCCGGGG
CYP24a1	AAGTGTGCCATTTACAACTC	GTTAACACTGTTCCTTTGGG

## Data Availability

The data presented in this study are available on request from the corresponding author.
